# Premature atherosclerosis in children with beta-thalassemia major: New diagnostic marker

**DOI:** 10.1186/s12887-017-0820-1

**Published:** 2017-03-09

**Authors:** Laila M. Sherief, Osama Dawood, Adel Ali, Hanan S. Sherbiny, Naglaa M. Kamal, Mohamed Elshanshory, Osama Abd Alazez, Mohamed Abd Alhady, Mohamed Nour, Wesam A. Mokhtar

**Affiliations:** 1 Pediatrics and Pediatric Hematology and Oncology, Faculty of Medicine, Zagazig University, Zagazig, Egypt; 2 Radiology Department, Zagazig University, Zagazig, Egypt; 3 Pediatrics Department, Zagazig University, Zagazig, Egypt; 4 Pediatric Department, Cairo University, Giza, Egypt; 5 Pediatric Department, Tanta University, Tanta, Egypt; 6 Biochemistry Department, Zagazig University, Zagazig, Egypt

**Keywords:** Beta-thalassemia, Carotid artery intima media thickness, Osteoprotegerin, premature atherosclerosis

## Abstract

**Background:**

Early vascular alteration, atherosclerosis and coronary artery disease have emerged as important cardiovascular complications among beta-thalassemia major (B-TM) patients. The aims of the current study were to assess the prevalence of premature atherosclerosis among our B-TM patients, and to investigate the diagnostic value of serum Osteoprotegerin assay as an early biomarker for atherosclerosis.

**Methods:**

This cross-sectional study was conducted at Hematology unit - Pediatric Department, Zagazig University Children Hospital- Egypt in the period from March 2014 to March 2015. A total of 115 children were enrolled in the current study; as sixty-five (65) children with beta thalassemia major aged 5–18 years, on regular blood transfusion regimen represented the patient group. While fifty (50) healthy children, with comparable age and gender, were assigned as control group. All participants were subjected to history taking, thorough clinical examination and laboratory investigations including; complete blood count, liver and kidney function tests, C- reactive protein, lipid profile, serum ferritin and serum Osteoprotegerin (OPG) assay. Also, carotid artery intima media thickness (CAIMT) was performed by duplex ultrasound for patients and controls.

**Results:**

Our B-TM patients were transfusion-dependent for as long as 8.5 ± 3.8 years with significantly higher serum ferritin levels (2490 ± 1579 ng/dl vs 83 ± 32 ng/dl, *p* = 0.001), C-reactive protein (5.7 ± 5.7 vs 0.9 ± 0.9), liver enzymes and bilirubin when compared to controls. Significantly higher serum triglyceride (128 ± 20 vs 101 ± 7 mg/dL, *p* = 0.009) and atherogenic index of plasma (0.45 ± 0.12 vs 0.22 ± 0.04, *p* = 0.001) were recorded in patients than comparisons. On the contrary, total serum cholesterol (116 ± 16 vs 143 ± 5, *p* < 0.001), low density lipoprotein-cholesterol (LDL-C) (44 ± 9 vs 73 ± 6, *p* < 0.001) and high density lipoprotein cholesterol (HDL-C) (39 ± 2 vs 61 ± 5, *p* < 0.001), were significantly lowered in patients versus normal peers. Carotid arteries intima media thickness (CAIMT) of both side were significantly increased for patients (Rt 0.62 ± 0.2 vs. 0.29 ± 0.07 mm, *p* = 0.001 & Lt 0.66 ± 0.17 vs 0.29 ± 0.05 mm, *p* = 0.001) when compared with healthy controls, and showed positive correlation with, serum triglyceride, atherogenic index of plasma, and serum Osteoprotegerin levels. ELISA assay of serum Osteoprotegerin (OPG) revealed significantly higher levels for thalassemia patients than matched healthy controls (427 ± 102 vs. 324 ± 126 pg/ml, *p* = 0.02). Of particular interest is the obvious positive correlation between OPG levels and CAIMT of both sides (Rt r 0.54, *p* = 0.001 &Lt r 0.479, *p* = 0.001) and also with serum triglycerides (r 0.374, *p* = 0.03).

**Conclusions:**

Subclinical atherosclerosis started prematurely in children with beta- thalassemia. Carotid artery intima media thickness represented a simple, accurate and non-invasivemodality for early detection ofatherosclerosis. It was correlated well with serum Osteoprotegerin; this finding highlighted the possible validity of OPG assay as an early predictor of atherosclerosis in thalassemia children.

## Background

Beta-thalassemia represents the commonest cause of hemolytic anemia in Egypt with carrier rate ranges from 9–10% [1]. Beta- thalassemia major (B-TM) patients usually present within the first two years of life with severe anemia requiring regular red blood cell transfusions [2]. Early vascular alteration, atherosclerosis and coronary artery diseases have emerged as important cardiovascular complications among B-TM patients [3].

Atherosclerosis is a slow progressive disease that may start at childhood [4]. In atherogensis, arterial wall morphological changes occur during a presumably long subclinical lag phase, and characterized by gradual thickening of the intima [5]. Beside the traditional diagnostic methods such as angiography and stress-testing [6], measurement of the intima-media thickness of the large arteries, especially the carotids, has emerged as one of the method of choice for determining the anatomical extent of arterial wall deterioration and for assessing cardiovascular risk [5]. Several investigators have recommended the clinical use of this technique for detecting subclinical (asymptomatic) atherosclerosis and for identifying subjects at high-risk [7–9].

Circulating markers of atherosclerosis are associated with increased vascular risk; one of the new biomarkers of atherosclerosis is Osteoprotegerin (OPG) [10]. Osteoprotegerin is a cytokine of the tumor necrosis factor (TNF) receptor superfamily and is classed as an osteoclastogenesis inhibiting factor [11]. In the endothelial cell, OPG is associated with Von-Willebrand factor within secretory granules called Weibel-Palade bodies. Upon stimulation with TNF or interleukin-1 in vitro, the OPG- Von-Willebrand factor complex is secreted in the surrounding growth medium. This endothelial activation by pro-inflammatory cytokines is one of the possible sources of circulating OPG in patients with active atherosclerosis [12]. Moreover, in view of the role of vascular smooth muscles in atherosclerosis and intimal calcification, it is likely that, they are the main source of increased circulating OPG noted in cardiovascular disease [13]. Data suggest that OPG is induced by atherosclerosis and may be upregulated as an incomplete compensatory response to the vessel insult, possibly thereby limiting vascular calcification [14]. Osteoprotegerin seems to prevent arterial calcification but is not able to reverse calcification once it has occurred [15].

As the maximal potential for prevention and reversibility of atherosclerosis would be expected by intervention at early subclinical stage of the disease, its early diagnosis in high-risk individuals should be a research priority. The aims of the current work are; to assess the frequency of premature subclinical atherosclerosis in Egyptian B-TM patients by determining their carotid artery intima-media thickness, to figure-out the associated clinical and laboratory risk factors, and to evaluate the validity of OPG assay as a new biomarker for early diagnosis of atherosclerosis in these high-risk population.

## Methods

### Patients

We carried out this cross-sectional, case–control study on sixty –five (65) Beta-thalassemia major patients aged 5–18 years old. They were on regular follow-up at the Hematology Unit of Pediatric Department, Zagazig University Children Hospital- Egypt during the period from March 2014-March 2015. Fifty (50) apparently healthy children with comparable age and gender distribution were enrolled as controls.

Thalassemia patients enrolled in the current work were on regular red cell transfusion regimen, 33 cases were transfused every 4 weeks, while 22 and 10 cases were transfused every 3 weeks and 2 weeks respectively to keep their target haemoglobin [16].

All subjects in our study were questioned for known risk factors for atherosclerosis, patients with history of smoking, hepatic, renal or cardiac diseases were excluded. Also those with diabetes mellitus or other endocrinopathies, other hemoglobinopathies, familial hypercholesterolemia or premature atherosclerosis in their families were also excluded.

The study protocol was approved by the research and ethical committee of Faculty of Medicine, Zagazig University and written informed consent was taken from parents or guardian of each participant.

### Methods

All eligible children (patients and controls) were subjected to; history taking with special emphasis on demographic characteristics, disease duration, frequency of transfusions, iron chelation regimen including type, dose, duration and compliance. Thorough clinical examination including anthropometric measures and all system evaluation were performed. Routine laboratory investigations for follow up thalassemia patients that included; complete blood count, liver function tests, renal function tests, serum ferritin, C-reactive proteins and hepatitis markers were taken.

Specific tests for evaluation of atherosclerosis were performed and included 12-h fasting lipid profile, serum Osteoprotegerin (OPG) assay, and carotid artery intima-media thickness. All blood sampling for patients were performed at the day before red blood cell transfusion.

Lipid profile was taken after at least 12 h overnight fasting, it was performed by endpoint method (colorimetric method) and included; total serum cholesterol, high density lipoprotein- cholesterol (HDL-C), serum triglyceride (TG). Low density lipoprotein –cholesterol (LDL-C) was estimated using *Friedewald and Levyformula* [17] by calculation of (TG)-(TG/5)-(HDL-C). Atherogenic index of plasma (AIP) is the ratio calculated as log (TG/HDL-C). Serum samples for assay of OPG were separated and stored at −20 C^0^. It was performed using Human OPG ELISA Kit, Boster Biological Technology Co., Ltd. USA.

Carotid artery intima-media thickness (CAIMT) measurements were performed for all participants by the same experienced vascular radiologist who was blinded to the clinical and laboratory details of the examined children. Duplex ultrasound B-mode and color-coded duplex sonography were performed using a (GE LOGIC P5) ultrasound system with a 12.0 MHz linear array transducer.

### Statistical analysis

All data were analyzed using SPSS 22.0 for windows (SPSS Inc., Chicago, IL USA) and MedCal 13 for windows (MedCal software bvba). Continuous variables were expressed as mean ± SD while categorical variables were expressed as number (percentages). Continuous variables were checked for normality using Shapiro-Wilk test. Independent student-*t* test was used to compare the normally distributed variables while Mann–Whitney U (MW) test was used to compare non-normally distributed variables between two groups. Categorical variables were compared by using Chi-square test (*X* ^*2*^). Pearson’ correlation coefficient and Spearman’ rank correlation were used to assess relationship between normally distributed and non-normally distributed variables respectively. All tests are two sided, and *p* < 0.05 was considered statistically significant.

## Results

Sixty-five B-thalassemia major (B-TM) patients were recruited in the current work, their mean age was 9.5 ± 3.7 years (ranged 5–18 years), and 57% of them were males. All of them were transfusion-dependent with illness duration ranged from 4–17 years (mean 8.5 ± 3.8 years), almost half of them (50.7%) were on every 4 weeks transfusion regimen, 34% were on 3 weekly regimen while for the remaining 15.3% cases every two weeks transfusion was needed to keep target hemoglobin. Deferiprone was used as iron chelation in 49% of them, 45% used Deferasirox while the remaining 6% were still using desferrioxamine as iron chelation therapy.

Results of B-TM patients’ clinical evaluations, routine and specific laboratory investigations were compared with those of fifty, age and gender, matched healthy children. Apart from height, no significant difference could be detected atdemographic or anthropometric measures between patients and controls as shown in Table 1. On the contrary, significant differences were documented when results of complete blood count and routine chemical analysis were evaluated as displayed in Table 2. B-TM patients had significantly higher white blood cell count and platelets but much lower hemoglobin concentration than healthy comparisons. Almost thirty fold rise in serum ferritin was reported (2490 ± 1579 ng/dl vs 83 ± 32 ng/dl, *p* = 0.001) for cases than controls. Significant rise in hepatic enzymes, total bilirubin and C-reactive protein were also revealed in patients group than healthy peers.

**Table 1:** Demographic data and Anthropometric measures of the studied groups

Character	Patients (*N* = 65)	Controls (*N* = 50)	Test	*p*-value
Age (years)				
Mean ± SD	9.5 ± 3.7	10.4 ± 3.7	MW = −0.348	0.729
Range	5 – 18	5 – 18		
Sex No (%)				
Male	37 (57%)	34 (68%)	*χ* ^2^ = 1.048	0.306
Female	28 (43%)	16 (32%)		
Residence No (%)				
Urban	25 (38.5%)	23 (46%)	*χ* ^2^ = 0.062	0.803
Rural	40 (61.5%)	27 (54%)		
Weight (Kg)				
Mean ± SD	27.5 ± 8.1	33.8 ± 10.8	t = −1.76	0.085
Range	14 – 45	19 – 60		
Height (cm)				
Mean ± SD	114.7 ± 21.8	132 ± 22	t = −2.15	0.038
Range	95 – 155	100 – 168		
BMI (Kg/m^2^)				
Mean ± SD	18.1 ± 2.2	21.5 ± 6.9	MW = 1.45	0.191
Range	16 – 21	16 – 33		

*MW* Mann Whitney *U* test, *χ* ^*2*^ Chi-square test, *p < 0.05* is significant, *t* independent Student *t*-test, *BMI* Body mass index

**Table 2:** Hematological and biochemical parameters of the studied groups

Parameter	Patients (*N* = 65)	Controls (*N* = 50)	Test	*p*-value
WBC (×10^3^/mm^3^)				
Mean ± SD	11.3 ± 4.6	7.3 ± 1.8	MW = 2.5	0.013
Range	9 – 18.3	5 – 9		
Hb (gm/dl)				
Mean ± SD	6.8 ± 0.9	12.6 ± 0.7	t = −17.5	<0.001
Range	5 – 8.3	11.8 –14.2		
PLT (×10^3^/mm^3^)				
Mean ± SD	478.4 ± 279.4	276 ± 77.6	MW = 2.2	0.030
Range	136 – 1428	170 – 405		
SGOT (IU/L)				
Mean ± SD	52.3 ± 39.4	30.4 ± 6.8	MW = 2.74	0.020
Range	9 – 160	20 – 40		
SGPT (IU/L)				
Mean ± SD	46.4 ± 38.4	29.7 ± 6.8	MW = 2.36	0.020
Range	8 – 155	19 – 38		
Total bilirubin (mg/dl)				
Mean ± SD	1.4 ± 0.6	0.6 ± 0.2	MW = 3.84	0.001
Range	0.6 – 2.8	0.4 – 0.9		
Direct bilirubin				
Mean ± SD	0.2 ± 0.1	0.2 ± 0.1	MW = −0.147	0.885
Range	0.1 – 0.5	0.1 – 0.4		
Total Proteins (gm/dl)				
Mean ± SD	6.1 ± 1.3	6.8 ± 0.8	t = −1.462	0.154
Range	4.5 – 8	5.2 – 8		
Urea (mg/dl)				
Mean ± SD	29.6 ± 6.9	27.5 ± 5.6	MW = −0.167	0.847
Range	21 – 41	15 – 37		
Creatinine (mg/dl)				
Mean ± SD	0.5 ± 0.3	0.4 ± 0.2	t = 0.341	0.766
Range	0.3 – 0.8	0.2 – 0.7		
CRP				
Mean ± SD	5.7 ± 5.7	0.9 ± 0.9	MW = 2.6	0.012
Range	1 – 21	0 – 2		
S. ferritin (ng/dl)				
Mean ± SD	2490 ± 1579	83 ± 32	MW = 4.7	<0.001
Range	653 – 8406	30 – 127		

*MW* Mann Whitney *U* test, *SGOT* Aspartate transaminase, *t* independent Student *t*-test, *SGPT* Alanine transaminase, *p < 0.05* is significant, *CRP* C- reactive protein

**Fig. 1:**
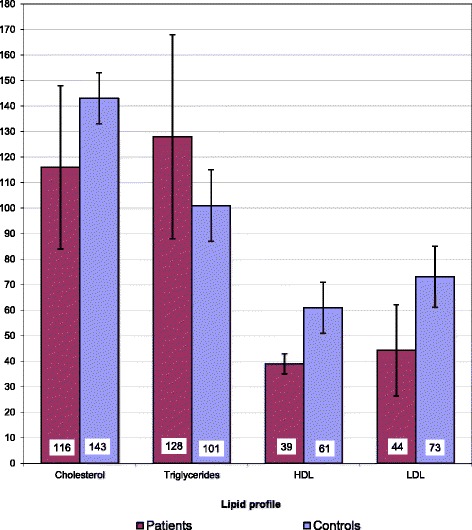
Lipid profile distribution of the studied groups: bar represent mean; Y-error bar represent 95% confidence interval of mean

Significantly higher serum triglyceride (128 ± 20 vs 101 ± 7 mg/dl, *p* = 0.009) and calculated atherogenic index of plasma (0.45 ± 0.12 vs 0.22 ± 0.04, *p* = 0.001) were recorded in patients than comparisons. On the contrary, total serum cholesterol (116 ± 16 vs 143 ± 5, *p* < 0.001), low density lipoprotein-cholesterol (LDL-C) (44 ± 9 vs 73 ± 6, *p* < 0.001) and high density lipoprotein cholesterol (HDL-C) (39 ± 2 vs 61 ± 5, *p* < 0.001), were significantly lowered in patients versus normal controls as displayed in Fig. 1.

**Fig. 2:**
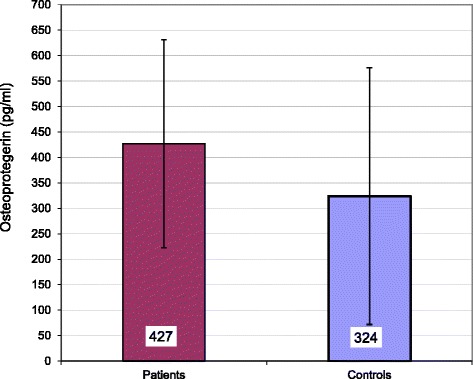
Serum Osteoprotegerin (pg/ml) distribution of the studied groups: bar represent mean; Y-error bar represent 95% confidence interval of mean

ELISA assay of serum Osteoprotegerin (OPG) revealed significantly higher levels for thalassemia patients than matched healthy controls (427 ± 102 vs 324 ± 126 pg/ml, *p* = 0.02) as expressed in Fig. 2. Of particular interest is the obvious positive correlation between OPG levels and CAIMT of both sides (Rt r 0.549, *p* = 0.001 &Lt r 0.479, *p* = 0.001) and also with serum triglycerides (r 0.374, *p* = 0.03) as shown among other parameter in Table 3.

**Table 3:** Correlations between Osteoprotegerin (OPG) and different parameters in the patients

Parameters	Osteoprotegerin (OPG)	
	r	*p*-value
Age	- 0.082	0.649
Duration of disease (years)	- 0.063	0.729
Body mass index (BMI)	+0.331	0.060
Serum ferritin	- 0.086	0.633
Serum cholesterol	- 0.205	0.253
High density lipoproteins (HDL-C)	- 0.263	0.140
Low density lipoproteins (LDL-C)	- 0.318^a^	0.071
Triglycerides (TG)	+0.374	0.032
Atherogenic index of plasma (AIP)	+0.263	0.140
C-reactive protein (CRP)	- 0.173	0.336
Right carotid artery intima media thickness (Rt. CAIMT)	+0.549	0.001
Left carotid artery intima media thickness (Lt. CAIMT)	+0.479	0.001
Aspartate transaminase (AST)	- 0.170	0.344
Alanine transaminase (ALT)	- 0.294	0.097

*r* correlation coefficient

^a^Pearson’s correleation coefficient

*p < 0.05* is significant

Duplex ultrasonographic Carotid arteries intima media thickness (CAIMT) of both side were significantly increased for patients (Rt 0.62 ± 0.2 vs. 0.29 ± 0.07 mm, *p* = 0.001 & Lt 0.66 ± 0.17 vs 0.29 ± 0.05 mm, *p* = 0.001) when compared with healthy controls as described in Table 4. These findings have pointed to the high frequency of atherosclerosis among thalassemia group. Documented positive correlation of CAIMT with serum triglyceride andatherogenic index of plasma have supported the previous data. Significant positive relationship between CAIMT and S. Osteoprotegerin (OPG) was also ascertained in our thalassemia patients as shown in Table 5.

**Table 4:** Radiological parameters of the studied group

CAMIT (mm)	Patients (*N* = 65)	Controls (*N* = 50)	MW	*p*-value
Right carotid artery (IMT)				
Mean ± SD	0.62 ± 0.20	0.29 ± 0.07	5.142	<0.001
Range	0.4 – 1.1	0.2 – 0.4		
Left carotid artery (IMT)				
Mean ± SD	0.66 ± 0.17	0.29 ± 0.05	6.609	<0.001
Range	0.4 – 1.2	0.2 – 0.4		

*M*W Mann Whitney *U* test, *CAMIT* Carotid artery intima media thickness, *p < 0.05* is significant, *mm* millimeter

**Table 5:** Correlation between carotid artery intima media thickness and different parameters of the patients

Parameters	Rt. CAIMT		Lt CAIMT	
	r	p	r	p
Age	+0.172	0.340	+0.083	0.646
Disease duration (years)	+0.195	O.276	+0.125	0.487
BMI	+0.123	0.494	+0.143	0.427
Serum ferritin	+0.200	0.264	+0.059	0.744
Serum cholesterol	−0.086	0.633	−0.087	0.631
TG	+0.275	0.121	+0.453	0.008
HDL-C	+0.164	0.361	+0.048	0.790
LDL-C	−0.235	0.188	−0.225	0.207
AIP	+0.155	0.388	+0.446	0.009
CRP	−0.115	0.525	−0.129	0.473
OPG	+0.411	0.018	+0.390	0.025
SGOT	+0.387	0.026	+0.003	0.985
SGPT	+0.307	0.082	+0.022	0.903

*CAMIT* carotid artery intima media thickness*, BMI* Body mass index*, r* Spearman’s rank correleation coefficient, *TG Triglycerides*, *p < 0.05* is significant, *AIP Atherogenic index of plasma*, *OPG* osteoprotegerin, *SGOT* Aspartate transaminase, *SGPT* Alanine transaminase, *CRP* C-reactive protein, *HDL*-*C* High density lipoproteins cholesterol, *LDL*-*C* Low density lipoprote

## Discussion

In the current study we tested the hypothesis that chronic hemolytic anemia may lead to vascular damage and premature atherosclerosis in B-TM patients. Our results documented significantly higher carotid artery intima-media thickness (CAIMT) of both sides among B-TM patients than matched controls (*p* < 0.001), a finding that provided evidence to the real risk of atherosclerosis for these patients.

As endothelial dysfunction and increased arterial thickness are important risk factors for the development of atherosclerosis [18], several studies have reported the measurement of arterial intima-media thickness as a good determinant of subclinical atherosclerosis [19–21]. Increased CAIMT have been described as a mirror of atherosclerotic burden, and a predictor of subsequent events including myocardial infarction and stroke [8, 19]. Because of its quantitative value, it has been used more and more in clinical trials [8], and noticeably trusted in detecting pre-clinical (asymptomatic) atherosclerosis in clinical setting [7]. Depending on the aforementioned advantages of this modality, our team and other researchers [19–21] have used this technique as a gold standard for early detection of atherosclerosis. Few studies have been performed on adults and adolescent patients with B-TM patients and showed significant increase in their CAIMT [21, 22] but to the best of our knowledge this work is the first to evaluate premature atherosclerosis in Egyptian B-TM children with this modality.

There are limited data concerning atherosclerosis risks in these patients, we evaluated clinical and laboratory parameters that may be relevant to vascular injury and atherogensis. Clinical characteristics didn’t show any difference from controls, or significant correlations with CAIMT. However, results of the hematologic and biochemical investigations of our B-TM patients displayed very peculiar metabolic model with; significant anemia, sky high increase in serum ferritin, and dyslipidemia which presented as high triglyceride and atherogenic index of plasma (AIP) but associated with low total cholesterol, LDL-C and HDL-C in patients as compared with comparisons.

Hemolytic anemia might be a predisposing factor for atherosclerosis by several mechanisms. First, erythrocyte release arginase enzyme during hemolysis coupled with the liberation of cell –free hemoglobin [23] contribute to dysregulated arginine metabolism with low arginine/ornithine ratio. These metabolic derangements limit the availability of arginine to nitric oxide synthase and lead to vascular dysfunction by disturbing the bioavailability of nitric oxide (NO) [24, 25]. Second, anemia was described as risk factor of hypertriglyceridemia due to its negative impact on extra-hepatic lipolytic activity [26].

Significantly high S. ferritin was found among B-TM patients in our series, this finding was in agreement with many previous researchers [27, 28]. Poor compliance to iron chelation was obvious among our patient (51%), represented an important and probably correctable cause of iron overload. High iron burden may increase patient’s risk for atherosclerosis by excess free radicle production [29, 30]. Moreover, *Mansi and Aburjai* documented positive correlation between S. ferritin and triglyceride level “an important predictor of atherosclerosis” [31]. Against our expectation, no significant correlation could be detected between CAIMT and S. ferritin, and similar result was previously documented by *Tantawy and his colleague* [21]. This data suggested that non-transferrin bound iron accumulation at cellular level with subsequent macrophage activation may be the triggering for development of atherosclerosis rather than high serum ferritin level [32].

Children with beta thalassemia are at increased risk of developing premature atherosclerosis because of dyslipidemia [21]. Lipid profiles have been described by several investigators but with conflicting results [26, 33–36]. Most of them including our team have shared common findings of lowered total cholesterol, LDL-C, HDL-C [27, 34–39]. Values from our B-TM patients and many other researchers showed elevated triglyceride levels (TG) [33, 34, 36–38], while others described TG levels as being not- significantly different from controls [35, 39]. CAIMT in the present study was positively correlated with S. TG and atherogenic index of plasma.

Dyslipidemia in thalassemia patients has been previously explained by several researchers who speculated different pathophysiologic pathways; plasma dilution because of anemia, accelerated erythropoiesis with excess cholesterol uptake by macrophages and histocytes of the reticuloendothelial system, defective liver synthetic function because of iron overload, macrophage activation with cytokine release and hormonal disturbance [33, 40, 41] while reduced extra-hepatic lipolytic activity might be responsible for the increased TG in B-TM patients [33].

It was surprising that our B-TM patients had early atherosclerosis despite low LDL-C, this observation is possibly due to oxidative change of LDL-C to “atherogenic LDL” by unbalanced oxidant- antioxidant milieu in thalassemia patients [42–45].

Atherogenic index of plasma (AIP) is a marker of atherogenicity since it is related directly to atherosclerosis; it is calculated as log (TG/HDL-C). Hypertriglyceridemia will increase the activity of hepatic lipase which results in HDL-C degradation with subsequent increased risk of coronary atherosclerosis [46]. In the present work, there was significant increase in AIP ratio for B-TM patients than for the healthy controls and also, it displayed positive correlation with CAIMT. These finding was in agreement with those by *Najajou et al. and Daniel et al.* who reported high predictive value of AIP for development of atherosclerosis [47, 48].

Rt. CAIMT showed significant correlation with SGOT (AST) but not with other liver enzyme, these findings might point to hemolysis in triggering atherosclerosis in our patients as hemolysis is the main source of elevated AST [49].

In the current study we had the chance to ascertain that Osteoprotegerin (OPG) was significantly raised in patients as compared with comparisons (*P* < 0.001), and to document the positive correlation that displayed between OPG, CIMT, and TG. Elevated OPG level was suggested; as a marker of arterial damage [50], as predictor of coronary artery diseases, [51] and overall cardiovascular morbidity and mortality [52], but as far as our knowledge our team was the first to investigate the relationship between atherosclerosis as expressed by CIMT and OPG in B-TM patients.

Results from two large cohort studies for the validity of OPG assay as a predictor of atherosclerosis and coronary artery diseases were supporting for its clinical application in predicting atherosclerosis in asymptomatic high-risk individuals [53], [54]. Positive correlation between OPG and CIMT observed among our B-TM patientshighlighted its importance as promising biomarker of subclinical atherosclerosis detection.

## Conclusions

Premature subclinical atherosclerosis was documented among Egyptian beta thalassemia major patients by evaluating their carotid artery intima media thickness (CAIMT). It was correlated well with dyslipidemia and serum osteoprotogrin, a finding that highlighted the possible validity of OPG assay as an early predictor of atherosclerosis in thalassemia children. However, multicenter wide scale research is warranted to evaluate OPG assay cut-off value, its sensitivity and specificity as reliable biomarker for diagnosis of atherosclerosis.

## Abbreviations

B-TM: Beta-thalassemia major.
CAIMT: Carotid artery intima media thickness
OPG: Osteoprotegerin
